# A Computer Mouse Using Blowing Sensors Intended for People with Disabilities

**DOI:** 10.3390/s19214638

**Published:** 2019-10-25

**Authors:** Hsin-Chuan Chen, Chiou-Jye Huang, Wei-Ru Tsai, Che-Lin Hsieh

**Affiliations:** 1 School of Computer Engineering, University of Electronic Science and Technology of China, Zhongshan Institute, Zhongshan 528402, China; chen_robin@foxmail.com; 2 School of Electrical Engineering and Automation, Jiangxi University of Science and Technology, Ganzhou 341000, China; 3 Department of Electronic Engineering, National Taipei University of Technology, Taipei 10608, Taiwan; 100404006@stud.sju.edu.tw; 4 Department of Electronic Engineering, St. John’s University, Tamsui, New Taipei City 25135, Taiwan; 100404058@stud.sju.edu.tw

**Keywords:** people with disabilities, mouse assistive technology, blowing control, re-triggerable monostable multivibrator

## Abstract

The computer is an important medium that allows people to connect to the internet. However, people with disabilities are unable to use a computer mouse and thus cannot enjoy internet benefits. Nowadays, there are various types of assistive technologies for controlling a computer mouse, but they all have some operational inconveniences. In this paper, we propose an innovative blowing-controlled mouse assistive tool to replace the conventional hand-controlled mouse. Its main contribution is that it uses microphones to induce small signals through the principle of airflow vibration, and it then converts the received signal into the corresponding pulse width. The co-design of software programming enables various mouse functions to be implemented by the identification of the blowing pulse width of multiple microphones. The proposed tool is evaluated experimentally, and the experimental results show that the average identification rate of the proposed mouse is over 85%. Additionally, compared with the other mouse assistive tools, the proposed mouse has the benefits of low cost and humanized operation. Therefore, the proposed blowing control method can not only improve the life quality of people with disabilities but also overcome the disadvantages of existing assistive tools.

## 1. Introduction

With the development of computer and internet technology, peoples’ lives have undergone major changes. Nowadays, people greatly rely on the use of computers and the internet in their work, information acquisition, shopping, or even entertainment, all of which deeply affect their daily lives. In computer-based devices, in general, a mouse is an important device to interact with the computer, and it is used as a pointer to access the graphical user interface (GUI) on the screen. However, according to the statistics of the World Health Organization, there are about 250,000~500,000 people with disabilities caused by accidents or illnesses every year. Their daily lives almost completely rely on family care, and it is more difficult for them to operate computers using conventional hand-controlled mice. Therefore, to improve the life quality of people with disabilities, it is necessary to design a humanized mouse assistive tool [1].

Apparently, people with disabilities may be unable to operate computers using conventional mice. People with disabilities, especially those with spinal cord injuries, can flexibly operate only above their neck, so most of the existing mouse assistive tools are implemented based on eyes, head, mouth, or blowing and sucking movements in order to provide the basic mouse functions [2,3,4]. The eye-tracking mouse is one of the popular and often-used mouse assistive tools, and it uses eye-movement tracking to control the cursor movement on a computer screen [5]. On the other hand, a head-controlled mouse uses sensors such as accelerometers and gyroscopes to detect head actions or captures information about head movements through computer vision [6]. Brainwave recognition, as a developing technology [7,8] allows people with disabilities to use their minds to control mouse movements. Furthermore, some other popular mouse assistive tools use mouth control technologies, which include the control of a sip and puff switch [9], bite operation [10], and mouth shape recognition [11]. All the mentioned mouse assistive technologies are described in detail in Section 2. However, in addition to their high manufacturing costs, there are some inconveniences and limitations in operating all the aforementioned mouse assistive tools.

Motivated to provide an easy way to access the computers and the internet to people with disabilities, we developed a blowing-controlled mouse to replace the conventional hand-controlled mouse. The main contribution of the proposed mouse is that it employs multiple electret microphones as blowing sensors and uses a technology that converts a blowing signal into a corresponding pulse width. Via the identification of the pulse width, various mouse operations such as a cursor movement, left/right click, drag and scroll can be completed. Unlike the other methods used in an assistive mouse, the proposed blowing-controlled mouse uses signal processing technology that converts a blowing signal into a pulse width, providing the benefits of a fast response and a lower implementation complexity compared with traditional digital signal processing. Since the proposed mouse only needs a slight blow from a user’s mouth and a small swing of a user’s head to operate for people with disabilities or even for paralyzed patients, it is relatively easy to control the computer. In addition, the proposed mouse can be applied to different computer operating systems without installing any driver; it thus possesses the feature of plug-and-play, because it can be set up by just connecting it to a USB (Universal Serial Bus) port.

This paper is organized as follows. Section 2 reviews the existing control methods of mouse assistive techniques. Section 3 introduces the proposed blowing-controlled mouse. Section 4 illustrates the implementation of the proposed mouse and provides the experimental results. Finally, Section 5 summarizes the features and performance of the proposed mouse.

## 2. Reviews of Mouse Assistive Technologies

At present, there are various mouse assistive tools on the market, and they use different control types, such as eyes-, head-, and mouth-based control methods. Table 1 shows the comparison of different mouse assistive technologies. In this section, we discuss the basic operating principles of various mouse assistive tools in detail.

### 2.1. Eye Control

In the past, the eye tracking was one of the popular control methods in devices intended for people with disabilities. Eye tracking is based on PCCR (pupil center corneal reflection) technology that tracks the eye movements and records the point of gaze related to the environment [12,13,14,15,16]. Such devices need a near-infrared light source projecting toward the user’s eyes, and they use a high-definition video camera to sense the light reflected from the user’s eyes. By using image processing and recognition, the cursor can move to where the user looks. Burger et al. [14] evaluated the suitability of an inexpensive eye-tracking device for the enhancement of user experience. Jiang et al. [15] pointed out that the research was proposed to survey players’ visual attention mechanisms of various interactive levels of mobile games’ interfaces under free-browsing and task-oriented conditions. Antunes et al. [16] proposed to use the eye tracking in a first-person shooter game as a mechanism. In summary, there have been many applications of eye control in computer games. However, people with disabilities easily feel eye fatigue after long periods of gazing, and the price for constructing such a mouse assistive tool is high due to the high amount of required equipment.

### 2.2. Head Control

This kind of mouse measures the head rotation angle by using an accelerometer and a gyroscope as sensors, and then it finds out the relationship between the head rotation angle and the mouse movement distance; however, some head-controlled techniques use a camera-based computer vision technology [17,18,19,20] to capture the user’s head movements to perform mouse functions. In addition, Rudigkeit et al. [17] proposed a human–robot interface that enables tetraplegics to control a multi-degree-freedom robot arm in real-time by solely using head motion, allowing them to single-handedly perform the easy manipulation of tasks. The results showed that the mapping of head motion onto robot motion was intuitive, and a smooth, precise, and efficient robot control was achieved. The operation of this head-controlled mouse is very easy, but it can cause people with disabilities, especially with anemia, to suffer dizziness and discomfort during long-time usage.

### 2.3. Mouth Control

The most direct method, mouth-based control, is designed for people with disabilities to control mouse movements with a joystick-like stick which they bite or by moving their tongue across the detection surface area of an optical device [21]. Additionally, the user initiates mouse button functions by blowing air into the device. Though this method is simpler and easily controllable, its manufacturing cost is high. Additionally, it is uncomfortable for the user’s mouth and can cause shoulder and neck aches during long-time usage.

The other type of mouth control is to blow and suck a pipe and then open or close a mechanical sip and puff switch [22] by using the airflow pressure to decide which mouse function to perform based on a number of particular cyclic blowing/sucking actions. However, it can be uncomfortable and inconvenient for people with disabilities to keep biting a pipe, and it can also be a little laborious.

In [23], the recognition of mouth shape was used as a control method. This mouse device uses a camera to capture images of mouth actions, and then it determines the mouth position and identifies the mouth shape as an opened mouth or a closed mouth to realize the mouse functions. The authors claimed that the detection accuracy of the opened mouth was higher than of the closed mouth when the mouth stayed still, but the detection accuracy decreased down when mouth kept moving [23]. For this mouth-controlled mouse, it is necessary to extract feature values from captured images of the mouth and build corresponding models for different mouth shapes. Therefore, this control method needs a pre-training before being used, and its identification accuracy can decrease when attempting multiple functions or when the mouth does not make a large and distinct action.

### 2.4. Brainwave Control

Brainwave control technology [24] has been developed recently, and the control method based on this technology requires a non-invasive EEG (electroencephalography) headset with many electrodes put on a user’s head to sense weak signals produced by the brain’s neurons through the scalp. Tanaka et al. [25] put forth an idea to develop an electroencephalogram-based control method for a mobile robot. The authors employed an algorithm to direct direction thinking and applied it to the direct control of a mobile robot, where the algorithm used wavelet transformation to initiate a time–frequency domain analysis. In the experiments of the EEG-based control for a mobile robot, the success rate of arriving at the desired positions was about 23%. Since the brainwaves of people differ, pre-training is necessary. The mouse device decides the correct mouse function by signal processing and computer learning. However, brainwave control has a large hardware and software complexity, and the system cost is very high. Additions, the identification rate of brainwave control is not yet good enough for the technology to move beyond the research stage.

## 3. Proposed Blowing-Controlled Mouse

**Figure 1:**
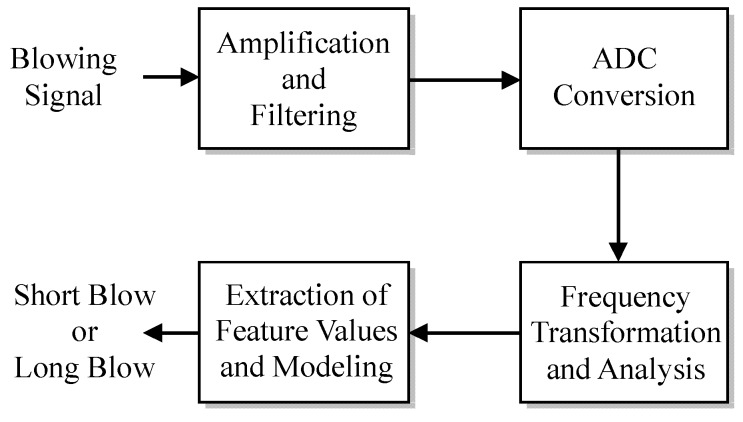
Conventional blowing signal processing.

Though the past studies have also used a microphone as a sensor to receive blowing signals, they have mainly focused on the digital signal processing of the blowing signal after amplification andanalog-to-digital conversion, which is similar to speech signal processing [26,27]. However, it is necessary to transform the blowing signal to the frequency domain by using a fast Fourier transform (FFT), then analyze and extract the feature values, and finally establish the models to determine whether the blow is long or short. The whole process shown in Figure 1 is more complicated. When there are multiple microphone inputs, a problem of mutual interference between the microphones may appear, and the hardware cost may increase.

**Figure 2:**
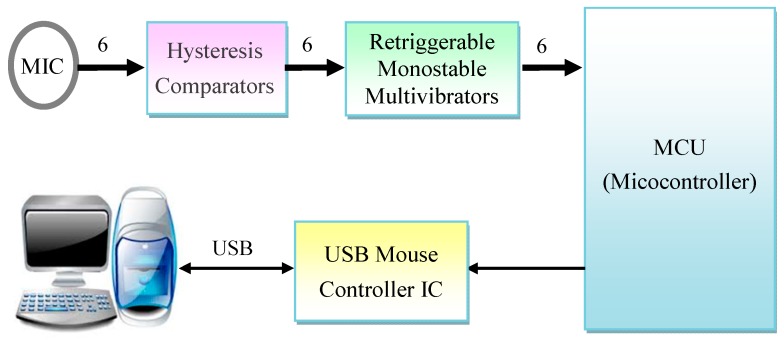
The architecture of the proposed blowing-controlled mouse.

In order to create a more convenient mouse assistive tool for patients with cervical spine injuries or limb defects, we developed a blowing-controlled mouse device based on airflow vibration to assist people with disabilities in manipulating mice. Figure 2 shows the system architecture of the proposed blowing-controlled mouse, in which six small electret microphones are used as sensing receivers of blowing signals; they represent the cursor’s movements, including up, down, left, and right, as well as left and right composite function keys. A hysteresis comparator and a re-triggerable monostable multivibrator (one shot) are used to complete the conversion of blowing signals to pulse widths. In addition, a microcontroller unit (MCU) is used to identify the position of the blown microphone and determine whether the blowing signal is long or short, and then it sends the pulses of movement coordinate or performs the key actions with the USB mouse controller chip. Finally, a USB mouse control chip with standard human interface device (HID) specification is used to communicate with the computer via a USB protocol to complete various functions of a mouse, such as cursor movement, left/right click, drag, and scroll. The blowing signal processing, the method of blowing position identification, and the basic working principle of the mouse control chip are described in detail in the following subsections.

### 3.1. Blowing Signal Processing

A technology without ADC (Analog-to-Digital Converter) that converts a blowing signal into the corresponding pulse width is proposed, and it includes the following three steps.

1. Based on the principle of airflow vibration, a small blowing signal is captured by the sense of an electret microphone.
2. Passing through a hysteresis comparator, many impulses are obtained in proportion to the time of the blowing signal.
3. A converted pulse width corresponding to the blowing impulses is generated by a re-triggerable one shot.

**Figure 3:**
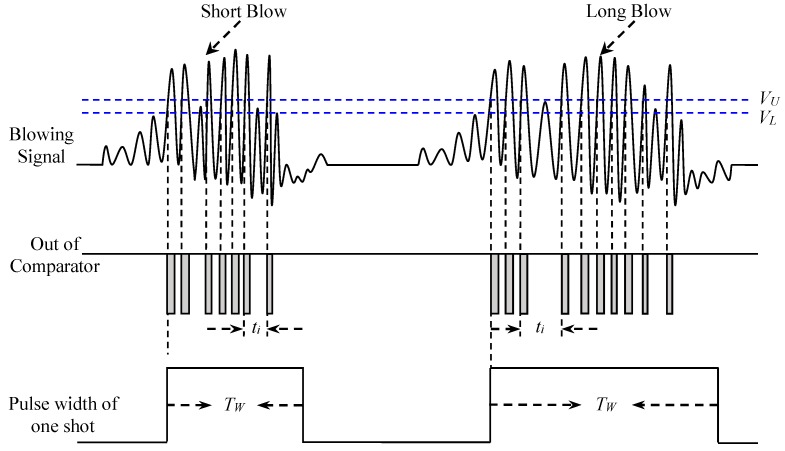
Conversion of blowing signal to pulse width.

Figure 3 illustrates the conversion of the blowing signal for both short and long blows; the converted pulse width is proportional to the blowing time.

**Figure 4:**
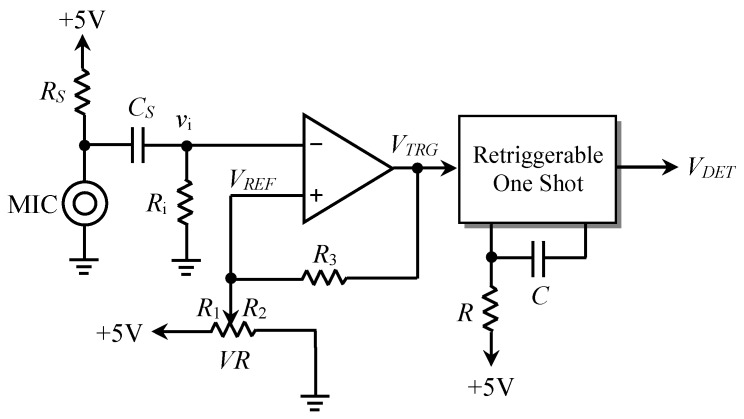
The circuit for blowing signal sensing and conversion.

In Figure 4, one of six sense and conversion circuits for capturing the blowing signals is presented. This circuit for blowing signal processing can be divided into three parts, namely a microphone sensor, a hysteresis comparator, and a re-triggerable one shot, they are described in detail in the following.

(1) Microphone sensor

A low-cost electret microphone is a type of electrostatic capacitor-based microphone. It has a stable dielectric material and high resistance, so it can be used as a receiving sensor to capture a blowing signal. During the microphone blowing, a weak signal is sensed via a microphone vibration caused by the airflow, and this small blowing signal is obtained by removing the DC bias by a coupling capacitor *C_S_*.

(2) Hysteresis comparator

To achieve digital trigger signals corresponding to a blowing signal, a hysteresis comparator is used to convert the blowing signal into a series of impulses (*V_TRG_*). Here, by changing a variable resistor (*VR*), the reference voltage *V_REF_* can adjust the sensitivity to sense the blowing signal, where *V_REF_* should be set to an appropriate value to obtain an optimum sensitivity. Since *VR* is equivalent to two resistors *R*_1_ and *R*_2_ in series connection, *V_REF_* can be expressed as:

(1) $$V_{REF}=\frac{R_{1}∥R_{2}}{(R_{1}∥R_{2})+R_{3}}\times (\pm V_{SAT})+\frac{R_{2}∥R_{3}}{R_{1}+(R_{2}∥R_{3})}\times 5$$

where *V_SAT_* is the saturation voltage of the comparator output and the hysteresis voltage *V_H_* of the hysteresis comparator is given by:

(2) $$V_{H}=\frac{2\times (R_{1}∥R_{2})}{(R_{1}∥R_{2})+R_{3}}\times V_{SAT}$$

(3) Re-triggerable one shot

The impulses generated by the comparator are discrete, and their intervals are not the same. Therefore, a re-triggerable one shot is required to make these impulses continuous. When these impulses enter into the re-triggerable one shot, an appropriate RC time constant needs to be selected such that the output pulse width *t_p_* of the one shot is two times greater than the maximum time interval between the impulses (*t_i_*), where *t_p_* can be expressed as:

(3) $$t_{p}\geq 2\times max\left\{t_{1},\;t_{2},\;\ldots \ldots \;t_{n-1}\right\}$$

Through the re-triggering operations, a continuous blowing pulse width (*T_W_*) corresponding to the blowing signal is given by:

(4) $$T_{W}=\sum_{i=1}^{n-1}t_{i}+t_{p}$$

where *n* denotes the number of impulses generated by a comparator, and it is proportional to the blowing time. Finally, the converted blowing pulse width is sent to the MCU controller for further processing.

### 3.2. Blowing Position Identification

**Figure 5:**
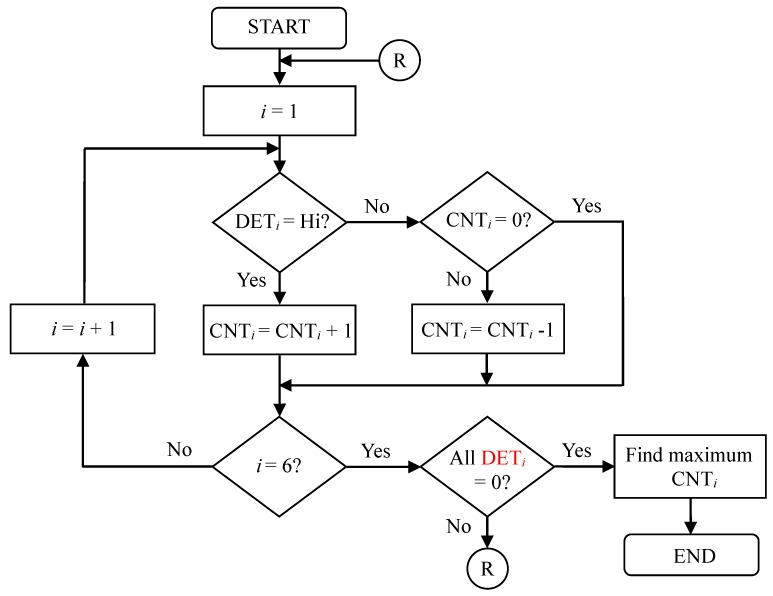
The identification algorithm for finding the maximum blowing pulse width.

Since there are multiple microphones on a blowing plate, when one of the microphones is blown into, the adjacent microphones may also sense the blowing signals. In this paper, we propose an identification algorithm to find the maximum blowing pulse width among all the microphones to reduce the mutual interference of multiple microphones; thus, the MCU controller can correctly determine the position of the blown microphone. Of course, there must be appropriate distance in the position arrangement of six microphones so that the identification accuracy can be improved. Figure 5 shows the flowchart of the identification algorithm for finding the maximum blowing pulse width, where *i* denotes the microphone number whose maximum value is 6, DET*_i_* denotes the blowing detection of the *i^th^* microphone, and CNT*_i_* denotes the blowing counter of the *i^th^* microphone. The MCU controller can use a timer interrupt to handle the detection and count of the blowing pulse width in the algorithm. The algorithm steps are as follows.

1. In the beginning, all microphones have a blowing detection (DET*_i_*) with a low level, and the initial value of their blowing counter variable (CNT*_i_*) is set to zero.
2. When a user blows into a particular microphone, the adjacent microphones may also receive a portion of the blowing signal.
3. In turn, the algorithm detects whether the DET*_i_* of each microphone is high. If it is high, the corresponding counter variable starts counting; otherwise, it decreases by one if the counter variable value is not zero.
4. The algorithm checks whether all the blowing detections (DET*_i_*) are returned to zero. If so, it stops the detection; otherwise, it repeats Steps (3)–(4).
5. Finally, the algorithm compares the blowing counters of all the microphones and finds the maximum blowing pulse width to identify the correct blown microphone position. At the same time, the MCU controller determines whether the signal is a long or short blow according to the counter value of the maximum blowing pulse width.

**Figure 6:**
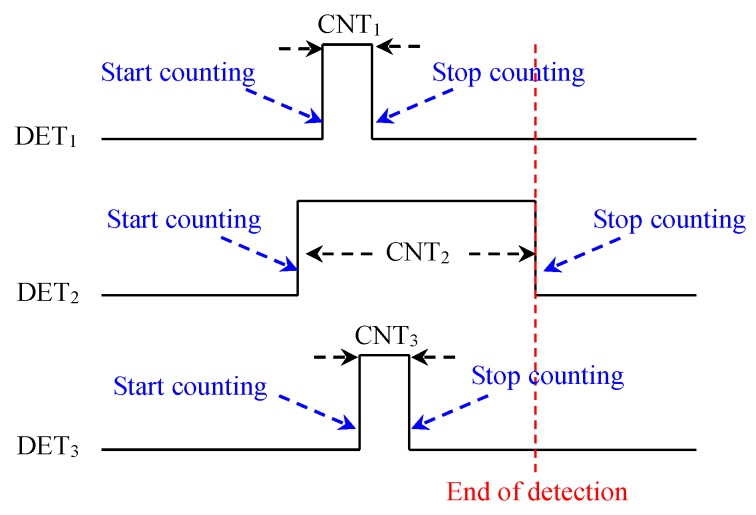
The principle of blowing position detection.

To more clearly understand the identification algorithm, a case study is provided: When a user blows into the second microphone, its detection signal DET_2_ turns to high from low, and then its counter (CNT_2_) starts counting until the DET_2_ turns to low again. At this moment, it is assumed that the first and third microphones may receive a portion of the blowing signal, which will cause their detection signals (DET_1_ and DET_3_) to be at high level, but their occurrence times will not necessarily be the same. Their corresponding counters (CNT_1_ and CNT_3_) also start counting until DET_1_ and DET_3_ turn to low. When all of the DET signals return to low, the current blowing detection is ended. According to the values of the counters (CNT_1_–CNT_3_), we can finally find that the value of CNT_2_ is maximum; therefore, the second microphone is considered as the main blowing target. Figure 6 illustrates the principle of the blowing position detection of the identification algorithm.

### 3.3. USB Mouse Control Chip

**Figure 7:**
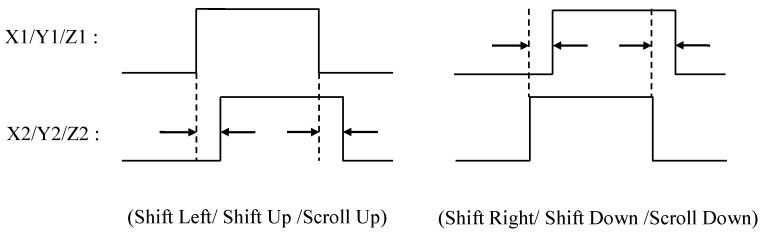
Timing of coordinate pluses corresponding to different mouse movements.

A USB mouse controller chip (for instance, TP8833) [28] with standard HID specification can be used to communicate with a computer via a USB protocol to complete various mouse functions, such as cursor movement, left/right click, drag, and scroll. Such a chip typically includes a USB serial interface engine (SIE) and a mouse functional unit; the SIE handles the transmission via a USB protocol, and the mouse functional unit provides an LED driver and several optical detectors to receive the photo-couple pulse signals caused by mouse movement. In the proposed mouse, due to the existence of the mouse controller chip, the MCU does not need to communicate with the computer as long as it converts the blowing signal into the corresponding input of the mouse button or the photo-couple pulses of the mouse movement. Additionally, it is fully compatible with various computer operating systems and has plug-and-play support without the need to develop any USB driver. This type of mouse controller chip is widely used in a mechanical wheel mouse or an optical mouse. In addition to providing the inputs of the middle, left, and right key switches, the chip also receives the photo-couple signals corresponding to the coordinates (X, Y, Z) where the mouse moves or scrolls. These photo-couple signals include the X1 and X2 pulses that indicate horizontal movement, the Y1 and Y2 pulses that indicate vertical movement, and the Z1 and Z2 pulses that indicate up and down scrolling. Figure 7 shows the timing relationship between the coordinate pulses; for instance, the cursor’s left movement is when the X1 pulse leads to the X2 pulse; inversely, when the X2 pulse leads to the X1 pulse that is the cursor’s right movement. Using the time difference between the two coordinate pulses in each direction, we can adjust the speed of cursor’s movement along that direction. After identifying the blowing position and the blowing pulse width, the MCU controller reproduces the coordinate pulses corresponding to the original mouse movement, - or sends the button inputs for the mouse controller chip to perform the click action of the left and right keys. Finally, the mouse controller chip directly communicates with the computer via the USB protocol; thereby, various functions of the conventional mouse can be implemented.

## 4. Implementation and Experimental Results

**Figure 8:**
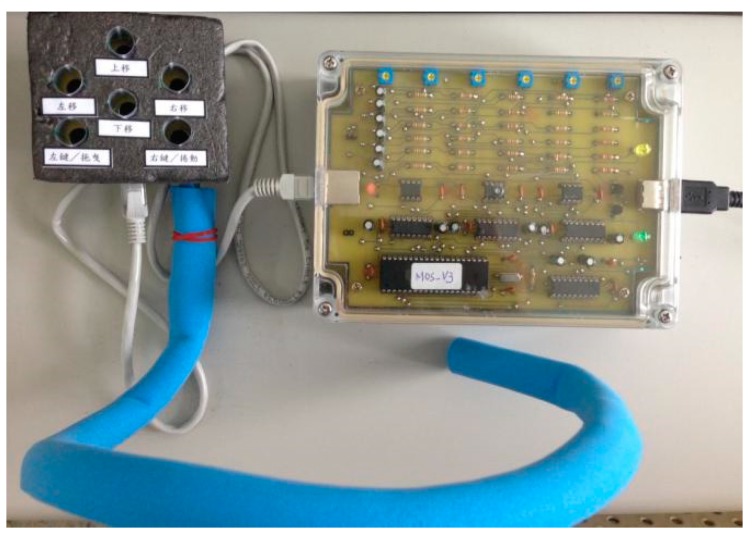
Image of the blowing-controlled mouse prototype.

**Figure 9:**
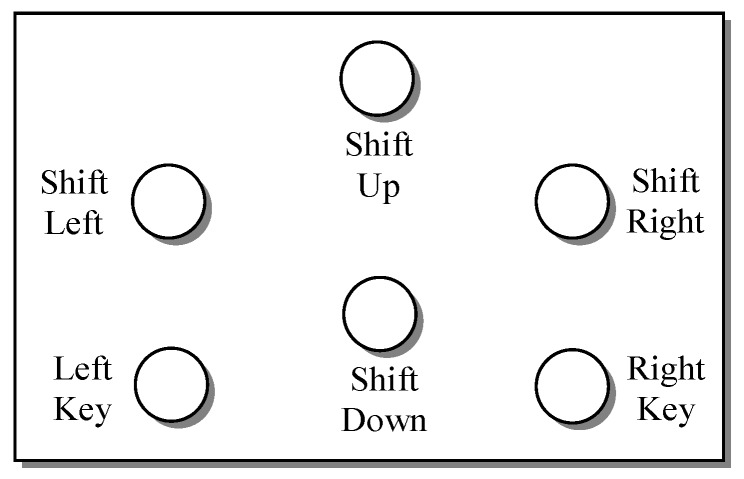
Function configuration of different microphones.

Figure 8 shows the practically implemented prototype of the proposed blowing-controlled mouse. In addition to the main control device connected to the computer via a USB port, the proposed mouse also includes a blowing panel mounted on the user’s neck. The function configuration of different microphones is shown in Figure 9, where the left and right microphones represent composite blowing function keys. A short blow into the microphone of the left key performs the clicking action and a long blow realizes the drag mode when it is combined with blows into the microphones labeled with different movement directions. Similarly, a short blow into the microphone of the right key performs the open action, and a long blow activates the scrolling mode. When a user blows into the up and down microphones under such a scrolling mode, the scrolling function can be performed.

### 4.1. Implementation Procedure

**Figure 10:**
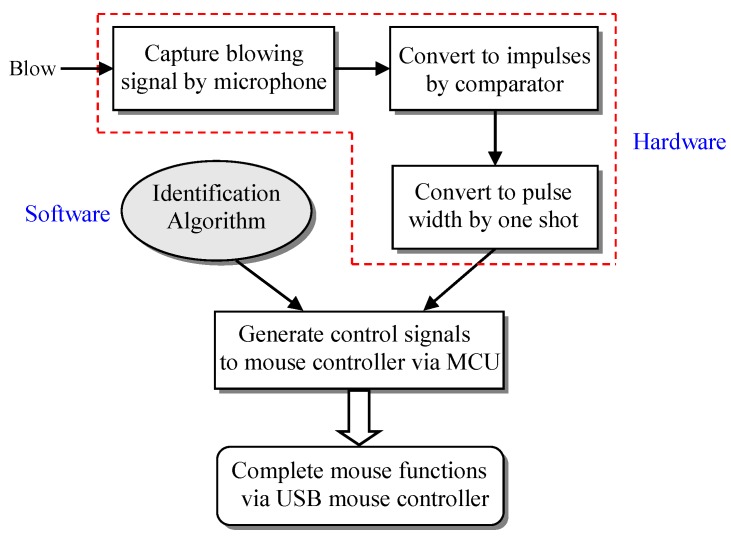
The implementation procedure of the proposed blowing-controlled mouse.

Figure 10 illustrates the implementation procedure of the hardware and software of the proposed blowing-controlled mouse. First, the blowing signal processing was realized in the hardware circuit, which included using the microphone to capture the blowing signal, converting the blowing signal into the corresponding impulses through the hysteresis comparator, and using a re-triggerable one shot to convert the impulses into the corresponding pulse width. To reduce the mutual interference of all the microphones, an identification algorithm to find the maximum blowing pulse width was executed by the MCU. After the identification, the MCU generated the control signals that were sent to the USB mouse controller such as coordinate pulses or the inputs of mouse buttons. Finally, the mouse controller chip directly communicated with the computer via the USB protocol and performed all the mouse functions.

### 4.2. Waveform Measurement

**Figure 11:**
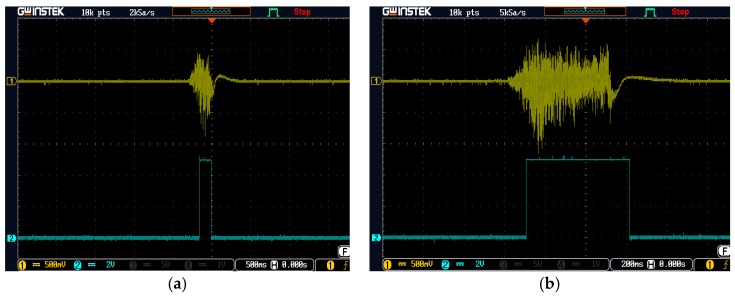
The waveforms of (**a**) a short blow, and (**b**) a long blow.

As shown in Figure 4, after the hardware blowing signal processing, the output of the re-triggerable one shot was converted into a pulse width that was proportional to the blowing time. Figure 11 demonstrates the measured waveforms of the blowing signals and their corresponding converted pulse widths. Figure 11a,b shows the waveforms corresponding to the short blow and long blow, respectively.

### 4.3. Sensitivity of Blowing Detection

**Figure 12:**
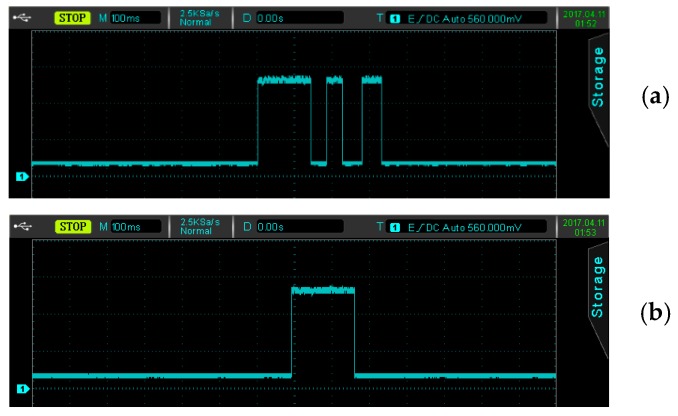
The pulse width output at (**a**) *V_REF_* (reference voltage) = 114 mV, and (**b**) *V_REF_* = 0.56 V.

The sensitivity of the blowing detection affects the accuracy of microphone identification, and thus, an optimal reference voltage *V_REF_* of the hysteresis comparator should be determined to facilitate further signal processing. In the practical experiments, the reference voltage *V_REF_* of the hysteresis comparator could be changed by adjusting the variable resistor *VR*, and the optimal reference voltage setting could be observed and found. In Table 2 and Figure 4, it can be seen that the output of one shot was as follows: *V_DET_* remained high when *V_REF_* was set too low (<0.5 mV), and *V_DET_* had hardly any pulse width output when *V_REF_* was larger than 0.98 V. When *V_REF_* was set at in the range 0.5–219 mV, the comparator generated a long-impulse-interval output at the end of a long blow, and then *V_DET_* had an intermittent pulse width output as shown in Figure 12a, which could easily cause a misjudgment. Therefore, by choosing a *V_REF_* value close to the range 0.22–0.83 V, once the microphone had been blown into, a complete pulse width output corresponding to the blowing signal (shown in Figure 12b) could be obtained.

### 4.4. Identification Rate

In this subsection, we evaluate the identification rate of the proposed blowing control method and compare it with mature speech recognition methods [29,30]. In general, the identification rate decreased with the increase in the distance between the sensor and signal source; as such, an appropriate distance is 3–4 cm. Table 3 shows that the proposed blowing identification method achieved an excellent identification rate of over 90% for the short blow and 85% for the long blow under the conditions without speech interference. The speech identification rate was also higher than 70%; the identification errors were mainly caused by the incorrectness of the semantic analysis or speaker’s inaccurate pronunciation. On the other hand, even when someone spoke alongside the user, the identification rate by using the proposed blowing identification was almost unaffected. However, the speech recognition was easily disturbed by environment sounds, and its identification rate immediately decreased below 60% when multiple voices appeared at the same time.

### 4.5. Evaluation of Mouse Operation

In the development process, the proposed blowing-controlled mouse was practically operated by different people by repeating the same tests. Table 4 shows the usage evaluation for different people and includes their age, gender, training time, operational time, and reaction (where the training time is the first practice time to learn how to operate the proposed mouse). Since everyone has a different mastery of blowing skills, some people need more practice time to learn how to blow continuous air to perform a correct long blow, and the average training time was about 5.8 min after an evaluation by ten persons. Therefore, regardless of age and gender, people can get start quickly after knowing the operational points. Because the operational habit of the proposed mouse is similar to that of a hand-controlled mouse and there is no need to blow hard nor to substantially swing one’s head, none of the users felt dizzy after a usage of about 30 min—even elders. Additionally, some people thought it was easy and effortless, with some even not feeling tired. In general, after more practice, people will be more familiar with the proposed mouse and then be able to use it smoothly.

As for the measurement of target acquisition time, we asked our subjects to to move the cursor from 5 cm around the target to the target through short blowing into the movement direction microphones, and we found that the average target acquisition time was about 4.43 s after 10 measurements in a fine-tuned mode. When the distance between the cursor and the target was larger—for example, from the rightmost of the screen to the left most of the screen—the average target acquisition time was about 4.7 s at a fixed speed of automatic movement through long blowing into the left movement microphone. In addition, in order to provide feedback to the users in operation, an LED or a buzzer can be added to indicate whether the blown microphone is sensed to remind users of its status.

The operational skill was very simple and user-friendly, as long as the user slightly blew against the microphone for operation. For instance, if a user wanted to move the cursor from the far-right side to the leftmost icon on a 27-inch computer screen, the user could slightly blow long into the left movement microphone, and then the cursor automatically shifted left; the speed of automatic movement was about 0.13 m/s. When the cursor approached the target icon, the user could shortly blow into any microphone to stop cursor movement. When there was a little position offset between the cursor and the target icon, the cursor could be fine-tuned to the target icon by short blowing into the up, down, left and right microphones. Additionally, the response time from blowing into the microphone to the moment of execution of the corresponding action was only about 100 ms. According to the presented mouse operation principle, a user is less likely to feel tired when manipulating the proposed blowing-controlled mouse, and the mouse can be easily manipulated by simple actions. Due to its high identification rate, as presented in Section 4.4, almost all of the microphones that are blown into can be quickly and accurately recognized, and the corresponding actions can be immediately performed.

### 4.6. Discussion on Results

As presented in Table 1 and described in Section 2, the existing mouse assistive tools adopt different control methods, but most of them have operational inconveniences and limitations. After profound analysis and research, we have found a simple control method that is easy to implement through blowing identification, especially by using our proposed technology of blowing signal to pulse width conversion, which can achieve better results. Since the control method of the proposed mouse significantly differs from the existing mouse control methods in construction and usage, it is difficult to compare the performance of our and other methods; therefore, Table 5 provides a comparison of different mouse assistive tools regarding their operational convenience, complexity, and price.

According to the experimental results provided above, as well as further analysis, we found that the sensitivity of blowing detection depends on the reference voltage *V_REF_* of the hysteresis comparator, which further affects the blowing identification rate. Namely, when *V_REF_* was set to an optimal value of about 0.22–0.83 V, the proposed blowing-controlled mouse could achieve an excellent identification rate of over 90% under conditions without speech interference; regardless of whether the signal was a short or long blow.

To sum up, the aim of the proposed blowing-controlled mouse is to help people with disabilities to easily use computers and the internet, helping them improve their life quality. Moreover, the proposed mouse can be widely promoted to people with disabilities because it has a low-cost implementation.

The novelty and contributions of this work can be summarized as follows:

(1) Hardware-based blowing signal processing without ADC is used to convert a blowing signal into a corresponding pulse width.
(2) The assistance of an identification algorithm to find the maximum blowing pulse width can reduce the mutual interference of multiple microphones, and the identification rate can thus be increased.
(3) The proposed technology requires no effort in operation and provides operational convenience, which makes it especially suitable for people with disabilities.

## 5. Conclusions

To overcome the operating drawbacks and limitations of conventional mouse assistive tools, a hardware-based blowing signal processing technology has been proposed in this paper to help people with disabilities, especially paralyzed people, to easily control a computer mouse. Using the software co-design of an identification algorithm, the proposed blowing-controlled mouse can be successfully equipped with various mouse functions, and it can achieve a stable and accurate identification rate even in the presence of other-sound interference. The experimental results showed that an identification rate of over 85% can be achieved. Moreover, compared with other mouse assistive tools, the proposed mouse has the advantages of low cost and user-friendly operation; thus, it can help people with disabilities to promote their life quality.

## Figures and Tables

**Table 1:** Comparison of different mouse assistive technologies.

Control Types		Technologies	Operational Convenience	Price	Disadvantages
Eye Control		Eye Tracking	Simple	Higher	Easy to cause eye fatigue
Head Control		Camera-based Computer Vision	Simple	High	Easy to cause dizziness for people with anemia
Mouth Control	Sip and Puff	Control of Sip and Puff Switch	Need of more exercises	Medium	Feel a little laborious, uncomfortable, and inconvenient
	Bite	Bite Combining with Blowing and Sucking Actions	Easy	High	It is uncomfortable for the user’s mouth, shoulder, and neck
	Mouth Shapes	Image Recognition	Need of pre-training	Higher	Mouth must perform a large and distinct action
Brainwave Control		Brainwave Recognition	Need of pre-training	Very Expensive	A large complexity of both hardware and software, and unsatisfactory identification accuracy

**Table 2:** Detection sensitivity dependence on the reference voltage value_._

*V_REF_*	Comparator Output	Sensitivity
<0.5 mV	Self-generated impulses	Too High
0.5–219 mV	Too long impulse interval at the end of a long blow	High
0.22–0.83 V	Suitable impulse intervals	Optimal
0.84–0.98 V	Less impulses for a short blow	Low
>0.98 V	No detected impulse	No Response

**Table 3:** Comparison of identification rates.

Situation	Speech Recognition	Blowing Identification	
		Short Blow	Long Blow
Without Speech Interference	72.0%	94.7%	90.6%
With Speech Interference	60.0%	92.3%	87.8%

**Table 4:** Usage evaluation for different people.

No.	Gender	Age	Training Time	Operational Time	Reaction
1	Male	11	4.5 min	25 min	No dizziness. Easy
2	Female	13	8 min	33 min	No dizziness. Not tired
3	Male	22	5 min	35 min	No dizziness. Not tired
4	Female	21	4.5 min	30 min	No dizziness. Easy
5	Male	33	4 min	28 min	No dizziness. Effortless
6	Female	38	9 min	32 min	No dizziness. Not tired
7	Male	40	3.8 min	25 min	No dizziness. Easy
8	Female	48	5 min	27 min	No dizziness. Easy
9	Male	55	5.5 min	30 min	No dizziness. Not tired
10	Male	75	8.5 min	28 min	No dizziness. Effortless

**Table 5:** Performance comparison of different mouse assistive tools.

Control Types		Operational Convenience	Complexity	Price
Eye Control		Simple	High	Higher
Head Control		Simple	High	High
Mouth Control	Sip and Puff	Need of more exercises	Low	Medium
	Bite	Easy	Low	High
	Mouth Shapes	Need of pre-training	High	Higher
Brainwave Control		Need of pre-training	Higher	Very Expensive
Proposed Blowing Control		Easy	Low	Low
